# Correlation of expression profiles between microRNAs and mRNA targets using NCI-60 data

**DOI:** 10.1186/1471-2164-10-218

**Published:** 2009-05-12

**Authors:** Yu-Ping Wang, Kuo-Bin Li

**Affiliations:** 1 Institute of Biomedical Informatics, National Yang-Ming University, Taipei, 11221, Taiwan; 2 Center for Systems and Synthetic Biology, National Yang-Ming University, Taipei, 11221, Taiwan

## Abstract

**Background:**

MicroRNAs (miRNAs) are small non-coding RNAs affecting the expression of target genes via translational repression or mRNA degradation mechanisms. With the increasing availability of mRNA and miRNA expression data, it might be possible to assess functional targets using the fact that a miRNA might down-regulate its target mRNAs. In this work we computed the correlation of expression profiles between miRNAs and target mRNAs using the NCI-60 expression data. The aim is to investigate whether the correlations between miRNA and mRNA expression profiles, either positive or negative, can be used to assist the identification of functional miRNA-mRNA relationships.

**Results:**

Predicted miRNA-mRNA interactions were taken from TargetScan 4.1 and miRBase release 5. Pearson correlation coefficients between the miRNA and the mRNA expression profiles were computed using NCI-60 data. The correlation coefficients were then subject to the Benjamini and Hochberg correction. Our results show that the percentage of TargetScan-predicted miRNA-mRNA interactions having negative correlation in expression profiles is higher than that of miRBase-predicted pairs. Using the experimentally validated miRNA targets listed in TarBase, genes involved in mRNA degradation show more negative correlations between miRNA and mRNA expression profiles, comparing with genes involved in translational repression. Furthermore, correlation analysis for miRNAs and mRNAs transcribed from the same genes shows that correlations of expression profiles between intronic miRNAs and host genes tend to be positive. Finally we found that a target gene might be down-regulated by more than one miRNAs sharing the same seed region.

**Conclusion:**

Our results suggest that expression profiles can be used in the computational identification of functional miRNA-target associations. One can expect a higher chance of finding negatively correlated expression profiles for TargetScan-predicted interactions than for miRBase-predicted ones. With limited experimentally validated miRNA-target interactions, expression profiles can only serve as a supplementary role in finding interactions between miRNAs and mRNAs.

## Background

MicroRNAs (miRNAs) were first identified in *Caenorhabditis elegans*. Since then more than 5,000 sequences have been found and annotated in many organisms [1]. MiRNAs are small non-coding RNA molecules regulating gene expression through various mechanisms [1-3]. Many biological processes, such as development, cell differentiation, and even diseases, have been associated with the activity of miRNAs [4,5]. Given that miRNAs function through binding to the 3' untranslated regions (UTRs) of mRNAs, computational algorithms, such as miRanda, TargetScanS and PicTar, have been developed to search potential miRNA target sites throughout a genome using perfect or imperfect base paring at potential interaction sites [6-8].

MiRNAs were initially reported to silence the target genes by interfering translation without reducing the expression levels of the target mRNAs [9]. However, subsequent studies proved that mRNA degradation can indeed be induced by miRNAs [10,11]. Moreover, microarray analyses provide evidence that the expression of miRNAs decreases the abundance of many transcripts carrying potential miRNA target sites [12].

With the extensive applications of expression profiling, microarray analysis on miRNAs has become a fast and effective approach to detect distinctive signatures for specific tissues or disorders [13,14]. In cancer research, the association between miRNAs and oncogene regulation has been reported and miRNA's involvement in cancers has also been identified through microarray experiments [15-18]. With the increased availability of miRNA microarray expression data, systematic investigation on the interactions between miRNAs and target genes using expression data could give us information on miRNA regulation. For example, a novel algorithm predicting miRNA targets, GenMiR++, has been recently developed using microarray expression profiles in addition to sequence matching [19]. To study the interactions between miRNAs and target genes, correlations between expression profiles of miRNAs and the target mRNAs in brain tumors have also been studied [20]. Instead of manually altering a miRNA's expression level, the brain tumor study focused on the primitive associations between endogenous miRNA levels and mRNA expression, which does not potentially lead to artificial influences on the underlying regulatory networks. Accordingly, more accurate effects of miRNAs on mRNAs could be measured by directly computing the paired correlations. However, the samples used in the brain tumor study were derived from a single tissue of origin, raising a question whether more underlying information about miRNA-mRNA interactions could be excavated when large-scale data are used.

In the current study, we ask the question whether the expression levels of the miRNA target genes show strong correlation with that of the miRNA itself. We used the miRNA and mRNA expression profiles of the NCI-60, a panel of 60 human cancer cell lines from several distinct tissues [21,22]. The hypothesis is that, assuming the mRNA degradation mechanism is involved in miRNA-target interactions, computationally predicted or experimental validated miRNA-target pairs should demonstrate negative correlations because of the degradation, whereas intronic miRNAs might be co-transcribed with their host genes thereby showing positive expression level correlations [23]. Although we have made comparisons between the prediction methods of TargetScan and miRBase, it is not our intention to compare the prediction accuracy between them. Firstly this cannot be done using the expression data alone and secondly such a comparison has been reported recently [24,25]. What we are trying to do in this work is to provide suggestion to users who want to assess the predicted target mRNAs using gene expression data. With the correlation analyses using the NCI-60 data, our results show that negative correlations in expression profiles are more likely to be found for TargetScan-predicted miRNA-mRNA interactions than for miRBase-predicted ones. This observation is consistent with an earlier report[19]. Positive correlation profiles were also found between intronic miRNAs and their host genes. Overall the results suggest that simultaneously profiling miRNA and mRNA expression could be informative when exploring the regulation of miRNAs and mRNAs.

## Results

### Available probes on miRNA and mRNA microarrays

**Figure 1:**
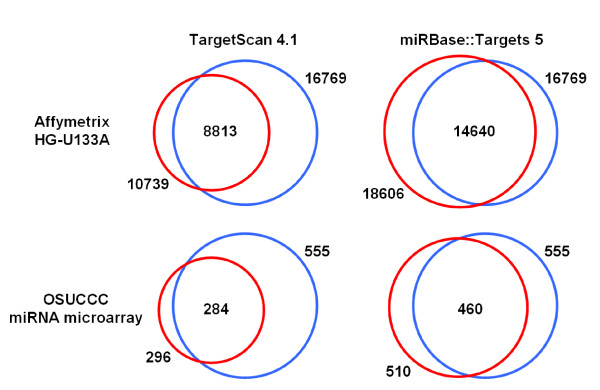
**The number of microarray probes used for computing the correlations of expression profiles**. The red circles denote the number of probes found in the predicted miRNA-target pairs. Two predicted data sources were used, including TargetScan 4.1 and miRBase::Target 5. The blue circles indicate the number of probes with at least two-fold intensity difference between the maximum and the minimum values across the NCI-60 samples. The intersection of the red and the blue circles is the number of probes used for the correlation analysis.

Filtering criteria (see Methods for more details) were applied to 59 NCI samples from nine tissues for all the expression profiles. The NCI-H23 cell line lacks mRNA data. The retained probes for correlation analysis on the microarray platforms should be those that appear in the downloaded miRNA-target data set and display adequate variability across the expression profiles. As illustrated in Figure 1, the red circles denote the number of probes whose corresponding targets or mature miRNAs can be found in the miRNA-target pairs predicted by TargetScan or miRBase, respectively. The blue circles indicate that 16,769 and 555 probes have at least two-fold difference in expression level between the maximum and minimum values among the 22,283 Affymetrix and 627 miRNA probes, respectively. The number in the intersection between the two circles is the set of probes used for correlation analyses.

### Correlation of expression profiles between miRNA and TargetScan predicted targets

**Figure 2:**
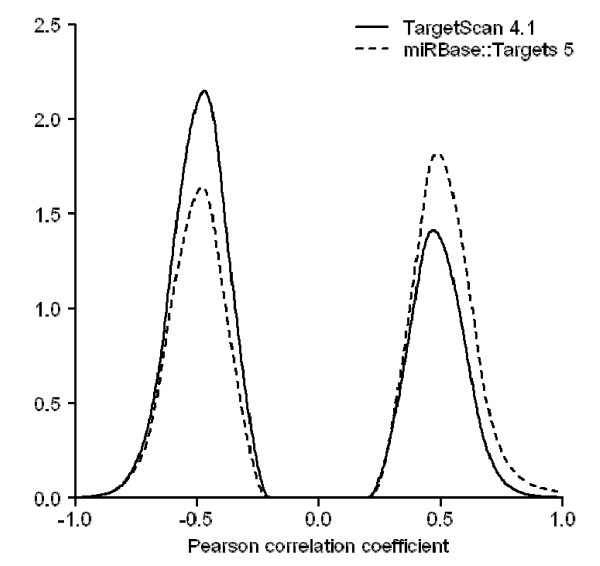
**The density plot of the positive and the negative correlation coefficients computed using miRNA-mRNA interactions predicted by TargetScan 4.1 and miRBase::Targets 5**. Pearson correlation coefficients were computed using both predicted miRNA-mRNA data. Significantly correlated pairs were selected after adjusting the *p*-values. The difference between the positive and the negative is not evident for the interactions predicted by miRBase::Targets, whereas the negative set is more dominant for interactions predicted by TargetScan.

The data set was taken from the TargetScan 4.1 web site. It contains 46,458 predicted pairs comprising 162 conserved miRNA families and 7,927 target genes. Using the filtering criteria (described in Method, also see Figure 1), we selected 284 miRNA probes and 8,813 Affymetrix probe sets to compute correlations. Among the 138,919 Pearson correlation coefficients and the corresponding *p*-values computed at the probe level, 2,976 probe-probe interactions, representing 1,389 predictions between 113 conserved miRNA families and 940 target genes, show statistical significance (Benjamini and Hochberg-adjusted *p *< 0.05). The percentages of positive and negative correlations are 39.28% and 60.72%, respectively. The density plot of the correlation is shown in Figure 2. As a random control test, we computed all the 2,502,892 correlations between 284 miRNA probes and 8,813 Affymetrix IDs, then randomly selected 138,919 values for 100 times to compare with those from the predicted pairs. The difference of the correlation coefficients between the TargetScan predicted pairs and the 100 random sets are all statistically significant (*p *< 2.2 × 10^-16^, Wilcoxon rank-sum test).

In the microarray data used in our study, a miRNA or a target gene could be represented by more than one probe thereby creating a problem of multiple probe-probe interactions. We found 664 miRNA-target pairs carrying multiple probe-probe correspondences that in turn lead to multiple correlation coefficients when comparing their expression profiles. For each of those 664 pairs, a standard deviation can be computed to estimate the variation of those correlation coefficients. Among the 664 pairs, 24 such standard deviations are found to be greater than 0.1, and only four pairs produce correlation coefficients with opposite signs. It suggests, in most cases, that probes representing the same gene indeed have similar expression profiles.

### Correlations of expression profiles between miRNAs and miRBase predicted target

The predicted miRNA-target pairs provided by the miRBase::Targets database are presented as interactions between individual miRNAs and target mRNA transcripts. The miRBase::Targets 5 contains 676,265 paired predictions for human, composed of 711 mature miRNAs and 34,525 Ensembl transcript IDs. As shown in Figure 1, 460 miRNA probes and 14,640 Affymetrix probe sets were used to perform correlation analysis. We obtained 3,575 significant *p*-values for the 293,176 correlations by using the Benjamini and Hochberg correction method. Those significantly correlated probe-probe pairs consist of 3,210 miRNA-target interactions, among which 303 miRNAs and 2,227 mRNA transcripts were found. As shown by the density plot in Figure 2, the negative correlations cover 46.18% of the significant pairs, comparing with 60.72% for the TargetScan predicted interactions. This observation is consistent with the earlier claims [19] that the TargetScanS-predicted target genes are more likely to be down-regulated owing to miRNA-mediated mRNA degradation. For all the 6,734,400 correlations, we also tested whether correlation coefficients from the predicted and randomly selected probe-probe pairs are different. The *p*-values from the 100 random tests indicate significant difference (*p *< 10^-6^, Wilcoxon rank-sum test).

As described earlier, certain miRNA-target pairs show multiple probe-probe correspondences. Among the 715 such miRNA-target pairs, 39 of them produce standard deviations greater than 0.1 for the multiple correlation coefficients. In four cases, correlation coefficients with opposite signs were found. The observation indicates that in most cases the expression profiles produced by different probes representing the same gene indeed resemble each other.

### Comparison between TargetScan and miRBase

The density plot shown in Figure 2 was prepared from the miRNA-mRNA interactions predicted from TargetScan and miRBase, respectively. Due to the different prediction strategy adopted by the two methods, we found 284 miRNA and 8,813 mRNA probes in NCI-60 data for TargetScan and 460 miRNA and 14,640 mRNA probes for miRBase. The uneven data size raises a question that whether the bias toward negatively correlated miRNA-mRNA for TargetBase predictions can be attributed to the algorithm design or simply to the differences in data sizes.

**Figure 3:**
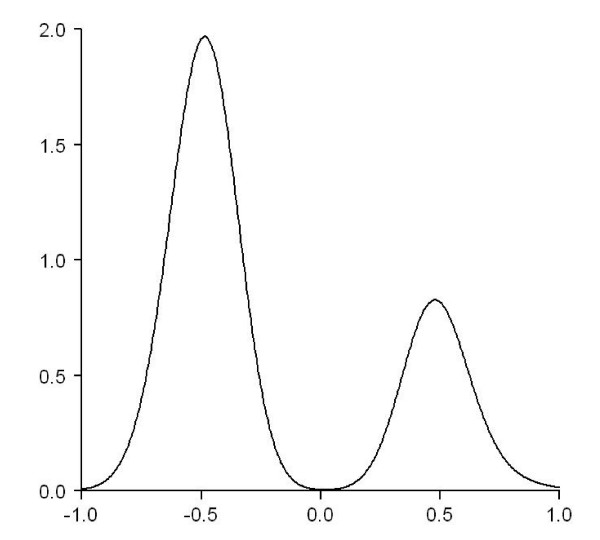
**The density plot of the correlation coefficients computed using the 17,777 common miRNA-mRNA pairs that were predicted by both TargetScan and miRBase**.

To address the concern, using the NCI-60 miRNA and mRNA expression data, we identified 17,777 miRNA-mRNA probe pairs that can be found in both the TargetScan and the miRBase predictions. We refer the 17,777 pairs as the common set. These pairs were then subject to the computation of correlation coefficients. After correcting the *p*-values of the correlation coefficients and keeping the 392 significant correlations, the resulting density plot is shown in Figure 3. Pairs predicted by TargetScan and by miRBase cannot be distinguished in Figure 3 because in the common set all data point come from the predictions made by both methods. Nevertheless it is clear that negative correlations occur more often than positive ones indicating that the expression levels of a miRNA and its target mRNA are more likely to be negatively correlated across the NCI-60 data than to be positively correlated, at least for the common set described here.

To compare the two methods of TargetScan and miRBase, one has to be able to distinguish the performance of both methods using some measurements. The measurement should produce different results for targets predicted by different method. For example, Baek et al[25] evaluated five target prediction methods by measuring the protein level change for targets predicted by each method. They also performed comparisons among the five methods using the best-scoring targets predicted by the respective approaches. Given that Figure 3 does not produce conclusive comparison between TargetScan and miRBase using a common probe set, in the following we will compare the distributions of negative and positive miRNA-mRNA correlations using three new experiments.

**Figure 4:**
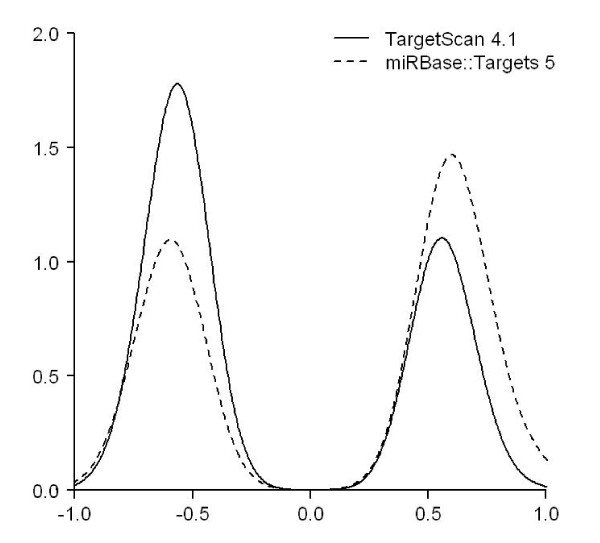
**The density plot of the 1,000 most positively or negatively correlated miRNA-mRNA pairs drawn from either TargetScan or miRBase datasets**.

To compare TargetScan and miRBase predictions, here we try to select equal number of statistically significant miRNA-mRNA pairs from both datasets. Using NCI-60 expression data, only those miRNA-mRNA pairs that have statistically significant Pearson correlation coefficients were retained. The correlation coefficients were ranked according to their absolute values. Only the top 1,000 pairs (from both datasets) with the most positive or negative correlation coefficients were used to draw the density plot shown in Figure 4. The figure shows the distribution of correlation coefficients of the 1,000 most correlated pairs for each of the two datasets. The distributions indicate that TargetScan-predicted miRNA targets tend to be more down-regulated when the comparison was performed using the same number, that is, 1,000, of the most correlated miRNA-mRNA interactions.

**Figure 5:**
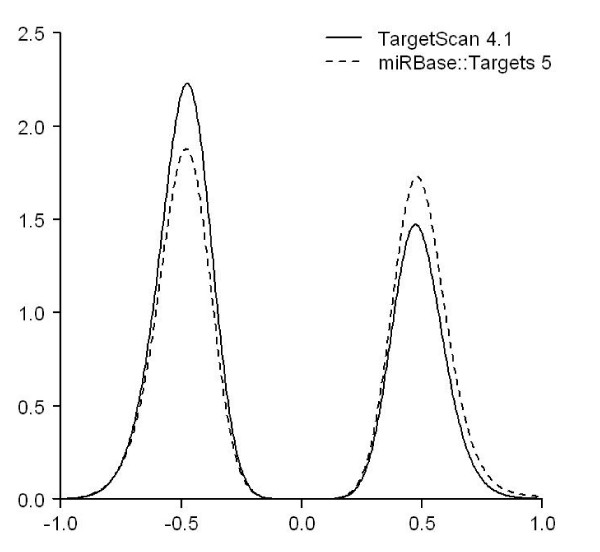
**The density plot showing the distribution of the correlation coefficients of 2,975 TargetScan-predicted and 3,027 miRBase-predicted miRNA-mRNA pairs**. **The predicted target mRNAs were obtained from a common set of miRNAs**.

Next we want to study the typical experimental scenario where target mRNAs are to be predicted off a particular set of candidate miNRAs that might have been selected using microarray or qPCR experiments. That is, in this experiment we had a common set of miRNAs and two sets of targets predicted by TargetScan and miRBase, respectively. Corresponding probes were identified. The filtering criteria were applied. The resulting pairs were subject to the computation of Pearson correlation coefficients using NCI-60 expression data. After *p*-value correction with the Benjamini-Hochberg method, pairs with *p *< 0.05 were considered as the statistically significant pairs. Their density plot is shown in Figure 5. For the 2,975 significantly correlated TargetScan-predicted pairs, 60.63% of them show negative correlations. For miRBase, 51.7% of the 3,027 significantly correlated pairs show negative correlation. This result indicates that, starting from a common set of miRNAs, expression profiles are more useful when assessing targets predicted by TargetScan than by miRBase.

**Figure 6:**
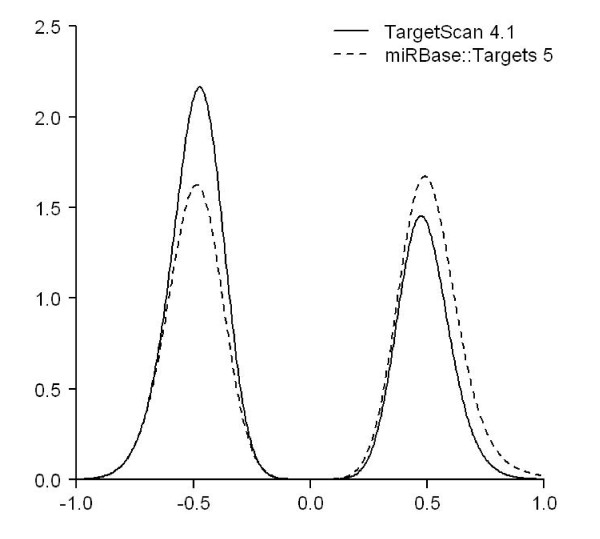
**The density plot showing the distribution of the correlation coefficients of 2,450 TargetScan-predicted and 1,890 miRBase-predicted miRNA-mRNA pairs**. **The predicted miRNA-mRNA interactions were obtained from a common set of mRNAs**.

Sometimes we want to identify miRNAs that have potential interactions with a set of candidate mRNA transcripts. Thus in this experiment we first selected common mRNAs in TargetScan and miRBase databases. Then we retrieved the predicted miRNA-mRNA interactions. The corresponding probe sets in NCI-60 expression data were then collected. The correlation coefficients were computed as described. Figure 6 shows the distribution of the significant correlation coefficients. The figure shows that 60.2% of 2,450 significantly correlated TargetScan-predicted pairs are negative, whereas 48.1% of 1,890 significantly correlated pairs are negative for miRBase. This result suggests that, given a common starting set of mRNAs, TargetScan predicted miRNA-mRNA interactions are more likely to show negative correlations and hence are possibly more suitable for the assessment using miRNA/mRNA expression profiles.

### Correlations of expression profiles for validated miRNA-target pairs

To investigate whether experimentally validated miRNA-target interactions show negative correlations of expression profiles, we computed the correlation coefficients for the validated miRNA-target pairs collected by TarBase [26]. The validated miRNA-mRNA pairs in Tarbase are divided into two classes according to their corresponding regulatory mechanisms: the translational repression and the mRNA degradation. The former class contains 96 pairs while the latter has 358 pairs. Among the 358 pairs listed by TarBase under the mRNA down-regulation or cleavage class, 274 of them have corresponding probes in the NCI-60 expression arrays. Similarly, probes in NCI-60 data can be found in 75 of the 96 translationally repressed miRNA-mRNA pairs.

Using Wilcoxon rank-sum test, the pairs involved in mRNA degradation show more negative correlations (*p *< 10^-8^) than those involved in translational repression. However, due to the multiple probe-probe correspondences, among the 349 (274+75) miRNA-target interactions we computed, 327 pairs have more than one correlation coefficient. Among the 327 pairs, the standard deviations of the correlation coefficients for 106 pairs are greater than 0.1, indicating that the profile correlation method is not reliable when applied to the validated data.

### Correlation of expression profiles for predictions made by GenMiR++

**Figure 7:**
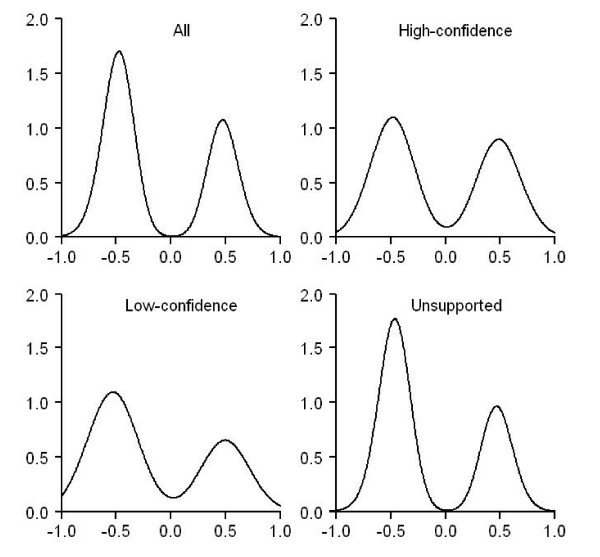
**The density plot of the correlation coefficients computed using miRNA-mRNA pairs predicted by TargetScanS and then filtered by GenMiR++**. The algorithm GenMiR++ provides a scoring system that determines the confidence level of the miRNA-mRNA interactions predicted by TargetScanS. There are three such levels: the high-confidence, the low-confidence and the unsupported. Using NCI-60 data, the distributions of Pearson correlation coefficients for the four categories specified by GenMiR++ (all, high-confidence, low-confidence and unsupported) are shown here. Only those correlations presenting Benjamini/Hochberg-corrected *p*-values < 0.05 are included. There are 412 such correlations in "All", 80 in "High-confidence", 41 in "Low-confidence" and 291 in "Unsupported".

GenMiR++ [19] was developed to identify functional miRNA targets. GenMiR++ classifies putative human miRNA targets that were originally predicted by TargetScanS into confident and unsupported ones using mRNA expression profiles. The expression data were obtained from 88 common normal and cancerous tissue samples [27,28]. Here we are to compute Pearson correlation coefficients for such miRNA-mRNA pairs to investigate whether GenMiR++'s confident and unsupported pairs show different correlations in terms of NCI-60 expression profiles. For the 5,572 miRNA-target interactions predicted by GenMiR++, a total of 16,388 miRNA-mRNA pairs at the probe-probe level were found in NCI-60 data. Among them, 3,467 are classified into the high-confidence class by GenMiR++, 4,421 the low-confidence and 8,500 the unsupported class. The computation of Pearson correlation coefficients and the subsequent Benjamini/Hochberg correction lead to 80, 41 and 291 significant correlations for the high-confidence, low-confidence and unsupported classes, respectively. Their density plot is shown in Figure 7.

Looking at the GenMiR++ prediction set as a whole (see the subfigure of Figure 7 that is labeled "All"), the density plot is similar to the one produced from TargetScanS's predictions, that is, negative correlations appear to be more dominant than positive ones. This result is expected because GenMiR++ takes TargetScanS's predictions as the input in the first place. What is worth noting is that the dominance of negative correlations seems to be more obvious in the unsupported class than in the high or low-confidence ones. While definite conclusion cannot be drawn from such a limited number of significant correlations (80 and 41 for the high and low-confidence classes, respectively), the observation might be attributed to two facts: (1) GenMiR++ and NCI-60 expression data come from different tissue samples; (2) For a given miRNA-mRNA interaction, GenMiR++ does not make its prediction using only the simple correlations in expression profiles. Instead, GenMiR++ considers all other predicted miRNA regulators of the mRNA using sophisticated inference algorithms [19].

Among the interactions that were validated by Huang *et al*.[19], the NCI-60 data show that the expression profile of let-7b is negatively correlated with the validated targets, SMARCC1 (*r *= -0.40, *p *= 0.06), CDC25A (*r *= -0.27, *p *= 0.29) and BCL7A (*r *= -0.37, *p *= 0.09). Furthermore, because miRNAs having identical seed regions are classified into the same family in the current released version of TargetScan 4.1, different putative targets of a miRNA family may actually interact with different members in the family. For this reason, we decided to see whether the experimentally validated let-7b targets are correlated with other members within the let-7 family. We found highly correlated let-7 member-target pairs, including let-7f/SMARCC1 (*r *= -0.49, *p *= 9.41 × 10^-3^), let-7a/CDC25A (*r *= -0.57, *p *= 9.54 × 10^-4^), and let-7c/BCL7A (*r *= -0.43, *p *= 3.84 × 10^-2^). Because a single miRNA recognition site could be targeted by miRNAs sharing the same seed sequence, we speculate that multiple miRNAs within the same family might simultaneously regulate the expression of the same target gene. As a result, the overexpression or suppression of different miRNA members in a miRNA family could lead to different changes in the expression levels of the target genes.

### Associations between intronic miRNAs and host genes

**Figure 8:**
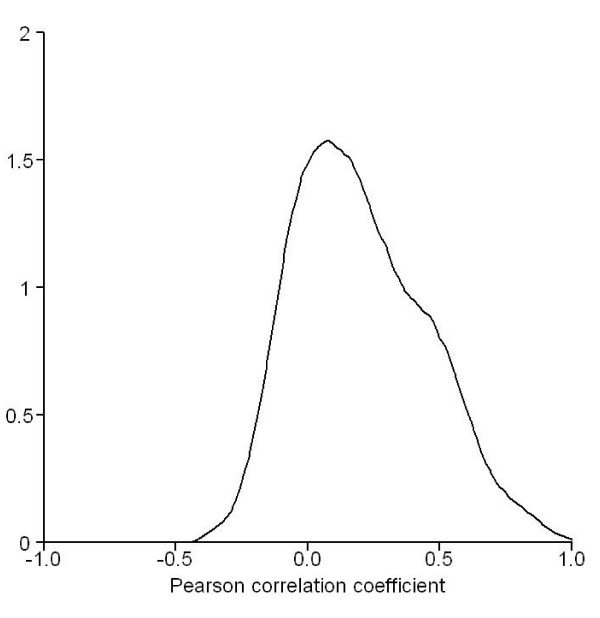
**The density plot of the correlation coefficients between intronic miRNAs and transcripts from their host genes**. The distribution of Pearson correlations between intronic miRNAs and their host genes is clearly biased toward to positive side.

We now ask whether intronic miRNAs have consistent expression profiles compared with their host genes. Here 139 probe-probe correlation coefficients representing 74 miRNA-host interactions were obtained. As shown in Figure 8, the distribution of those correlation coefficients is biased toward the positive correlation. Moreover, 41 significantly correlated pairs were found (Benjamini and Hochberg-adjusted *p *< 0.05), representing 25 interactions between 25 intronic miRNAs and 18 host genes. All of them are positively correlated (Table 1). This result suggests that the transcriptional regulation of intronic miRNAs is, at least in some cases, similar to that of the mRNAs from the same host genes.

**Table 1:** Intronic miRNAs and their corresponding host genes show significantly correlated expression profiles.

Intronic miRNA	Host gene	Correlation coefficient
hsa-mir-335	MEST	0.851
hsa-mir-342	EVL	0.811
hsa-mir-126	EGFL7	0.742
hsa-mir-452	GABRE	0.639
hsa-mir-26a-2	CTDSP2	0.616
hsa-mir-93	MCM7	0.584
hsa-mir-128a	R3HDM1	0.548
hsa-mir-27b	C9orf3	0.536
hsa-mir-152	COPZ2	0.526
hsa-mir-224	GABRE	0.529
hsa-mir-211	TRPM1	0.522
hsa-mir-26b	CTDSP1	0.520
hsa-mir-25	MCM7	0.481
hsa-mir-15b	SMC4	0.479
hsa-mir-106b	MCM7	0.475
hsa-mir-23b	C9orf3	0.469
hsa-mir-24-1	C9orf3	0.461
hsa-mir-218-2	SLIT3	0.461
hsa-mir-489	CALCR	0.425
hsa-mir-218-1	SLIT2	0.406
hsa-mir-425	DALRD3	0.406
hsa-mir-7-1	HNRPK	0.383
hsa-mir-191	DALRD3	0.381
hsa-mir-26a-1	CTDSPL	0.360
hsa-mir-16-2	SMC4	0.354

Among the correlations between intronic miRNAs and the host genes, the significant correlations are all positive.

## Discussion

In this study we investigated whether interactions between miRNAs and mRNAs are discernible as expected when computing correlations between miRNA and mRNA expression profiles. We first examined the pairs of miRNAs and predicted targets from TargetScan 4.1 and miRBase::Targets 5, respectively (Additional files 1 and 2). Pearson correlation coefficients were computed using both data sets. It is not surprising that the majority of the correlation coefficients are not statistically significant possibly due to that the true positive discovery rate of the two prediction programs cannot be accurately estimated in spite of the constant improvement of the algorithms. Besides, miRNA-target interactions that are involved in the repression of protein synthesis may not result in considerable alterations in the transcript expression level, hence leading to uncorrelated expression profiles. The results produced by different target prediction algorithms often diverge greatly by producing different sets of targets [29].

Figure 4 shows an experiment where equal number of statistically significant miRNA-mRNA pairs was selected from both TargetScan and miRBase. Here the statistical significance means that the adjusted *p*-value of the correlation coefficients in terms of the expression profiles between a miRNA and an mRNA is smaller than 0.05. In other words, a statistically significant miRNA-mRNA pair is the one who has a fairly negative or positive correlation. Given the condition, the figure shows that TargetScan predicted miRNA-mRNA pairs are more likely to present negative correlations. In Figures 5 and 6, we demonstrate that using a common set of miRNAs or mRNAs to start off the prediction, the percentage of TargetScan predicted miRNA-mRNA pairs having negative correlation is higher than that of miRBase predicted pairs.

For experimental biologists, the implication is as follows. If you were to assess whether a predicted miRNA-mRNA relationship is functional or not using negative correlation in expression profiles, for TargetScan predicted pairs, you are more likely to find supporting experimental evidence that their expression profiles are indeed negatively correlated. For miRBase predicted pairs, it is slightly less likely that your miRNA-mRNA pairs will show negative correlation thereby giving you a hint that the pairs are not functional. Our conclusion is consistent with a previous statement made by Huang et al[19] that "*interactions predicted by TargetScan are more likely to lead to mRNA degradation rather than translational repression*". A possible explanation for this observation is, compared with the miRanda algorithm used in the miRBase, that the latest TargetScan has incorporated more structural features to achieve higher predicting accuracy [30,31].

A Bayesian algorithm GenMiR++[19] claims that paired expression profiles of miRNAs and mRNAs can be used to differentiate functional and non-functional miRNA-target interactions. The algorithm produces a score for each TargetScanS-predicted miRNA-target pair. This score is then used to classify the interactions into three categories, the high-confidence, low-confidence and unsupported pairs. Using NCI-60 data and the interactions classified by GenMiR++, we ask the question whether high-confidence miRNA:target interactions indeed show more negative correlation in their expression profiles. Our result shows that, considering the high-confidence, low-confidence and the unsupported GenMiR++'s predictions as a whole, no considerable difference can be seen between the predictions made by GenMiR++ and by TargetScanS (see Figure 2 and 7). This is expected because GenMiR++ takes TargetScanS's results as the input data source (Additional file 3).

Because the tissue samples used to generate the microarray expression data adopted by GenMiR++ [27,28] are different from those used in NCI-60 experiments, experimental biases could arise due to such differences in data source. For example, the interactions between let-7b and its targets with high GenMiR++ scores have been experimentally validated by GenMiR++ authors [19]. Nevertheless in NCI-60 expression data, these experimentally validated pairs do not show significant negative correlations. On the other hand, the target genes of let-7b are found to be negatively correlated with other members of the let-7 family instead. Because all let-7 miRNA members share the same seed region, we speculate that more than one member in let-7 miRNA family may simultaneously regulate the expression of the same target gene. The overexpression or suppression of a family member could thus alter the expression level of the target gene. Furthermore, the seed regions of miRNAs are believed to be more important than the minor differences of nucleotides within the non-seed regions when targeting the 3' UTRs of mRNAs [8]. Many studies have also demonstrated the impact of structural factors other than sequence pairing when miRNAs recognize their targets [30,32,33]. Hence, we anticipate that a gene targeted by a miRNA could be regulated by more than one miRNA in the same family under different biological environments or conditions.

In addition to the putative miRNA-target interactions, we also tested whether Pearson correlation coefficients can be used to characterize miRNA-mRNA relationships, including the one between miRNA and validated targets and that between intronic miRNAs and their host genes. For the experimentally validated data, we tested the correlations of the paired expression profiles using miRNA-target pairs listed in TarBase (Additional file 4). Between the translationally repressed and post-transcriptionally downregulated target genes, the difference of the Pearson correlation coefficients in expression profile is significant. However we did not find significant negative correlations between miRNAs and their degraded targets. We propose the following explanations. (1) For a validated miRNA-target pair, there exist multiple correspondences between the miRNA and mRNA microarray probes. Not all mRNA array probes represent the transcripts having the miRNA recognition sites. (2) The miRNAs and target genes listed in TarBase are not updated according to the latest annotation, causing uncertainty when looking for corresponding probes on respective microarray platforms. (3) Most of the experimentally supported miRNA-target interactions leading to mRNA degradation were validated by a small number of research articles [12,34,35]. Those studies relied on mostly indirect evidences using microarray or real-time RT-PCR. (4) It has been suggested that expression or inhibition of miRNA targets could be tissue-specific [2,4]. Because our data are limited to the NCI-60 panel that are derived from specific cancer cells, associations between miRNAs and mRNAs might be overlooked if their relation can only be detected in a single tissue that is not included in NCI-60.

The positive correlations in our data reveal different relationships between miRNAs and genes, for example, the co-expression of intronic miRNAs and their host genes. As expected, we did not find any significant negative correlations between an intronic miRNA and transcripts from it host gene (Figure 8). Furthermore, 25 pairs of miRNAs and their host genes are found to be positively correlated. (Table 1, Additional file 5), among them the correlated expression of mir-126/EGFL7 and that of mir-342/EVL have been shown to play important roles in gene regulation of cancers [36,37]. At present, it is still unclear that whether a miRNA originating from non-coding regions could regulate its own host gene. Some hypothetical models have been proposed to infer the regulatory control of intronic miRNAs and their protein-coding host genes [38]. Those hypotheses would become another research subject in addition to investigating the connections between miRNAs and their target genes.

## Conclusion

Computing correlations between miRNA and mRNA expression profiles gives us an opportunity to study the effects of gene expression between miRNAs and their target genes. Our results suggest that microarray expression profiles could be used to assist the computational identification of functional miRNA-target associations. Expression profiles are more useful when assessing miRNA targets predicted by TargetScan than by other prediction methods. Using TarBase, we did not find significant negative correlations for the experimentally validated miRNA-target interactions. Because our analysis was only performed on the known and predicted miRNA-mRNA interactions, some significantly correlated probe-probe pairs might be ignored in our work due to the lack of information for their miRNA-target relationships. Those pairs might represent true miRNA-target interactions, the indirect miRNA-involved regulation, or just the coincident similarity in expression profiles. With the limited knowledge of miRNA-mediated gene regulations, systematic strategies to uncover true miRNA-target interactions by using expression profiles remain a challenge.

## Methods

### miRNA and mRNA microarray data

The NCI-60 mRNA microarray data were downloaded from ArrayExpress database , accession number E-GEOD-5720. The data were collected using the Affymetrix GeneChip^® ^HG-U133A platform [22]. The miRNA microarray data and the array design (Ohio State University Comprehensive Cancer Center, OSUCCC, version 3.0) can also be downloaded from ArrayExpress, accession number E-MEXP-1029. The miRNA array contains 627 probes from the two arms of selected human miRNA precursors. The mRNA data were normalized using the GCRMA algorithm [39], whereas the miRNA data were normalized according to the method described previously [21]. Both the miRNA and mRNA expression intensities were then transformed in logarithms of base 2. To investigate if spatial biases caused by autocorrelation exist in the miRNA microarray experiments, we applied an autocorrelation analysis using methods described in a previous report [40]. The next step is to determine the number of probes that are to be used for computing correlations. To ensure the quality of correlation tests, probes were selected using the following criteria. First, for mRNA, a probe set must have at least one corresponding target gene. Similarly, a miRNA probe should match one mature miRNA sequence stored in the prediction databases. Second, the difference between the highest and the lowest intensity values must be at least 1.0, meaning the range of variability among NCI-60 samples is at least two fold. Filtering out probes with low variability can reduce the chance of correlations from noisy values.

### miRNA and their targets

Two web databases of miRNA target prediction were used in this research. One is the miRBase::Targets (release 5) database , which uses the miRanda algorithm to predict miRNA targets [6,31]. Because the predicted targets are showed in the form of Ensembl Transcript IDs, we retrieved the corresponding Affymetrix probe set IDs via the BioMart website . The other database is the TargetScan (release 4.1) , which provides the prediction results computed by the TargetScanS algorithm [8,30,41]. In TargetScan, the predicted targets are presented as gene symbols, some of which are not HGNC-approved. The RefSeq IDs of predicted target genes are also retrieved. To correctly map target genes to microarray probe set IDs, we then accessed the Affymetrix website  to obtain the detailed HG-U133A annotation [42]. Because the miRNA annotation is continuously updated, to unambiguously mapping miRNA probes to mature miRNAs, we kept the sequence information of miRNAs provided by the target prediction web sites. Combining the oligo probe sequences of the OSUCCC miRNA microarray retrieved from the ArrayExpress, the annotation of the oligo probes were obtained by running BLAST against mature miRNAs (Additional file 6).

Unlike databases offering putative targets of miRNAs, the TarBase  collects the experimentally validated miRNA-target pairs [26]. We use TarBase to test whether the expression levels of the true miRNA-target pairs show correlations. The current version of TarBase is version 4. Because certain names of miRNAs and target genes provided by TarBase are not updated, we needed to update them using the latest microarray annotations.

To test whether using the correlation information of miRNA and mRNA expression profiles helps in the prediction of miRNA targets, we also downloaded the TargetScanS result that has been further processed by GenMiR++ [19]. In their result, 6,387 TargetScanS-predicted miRNA-target pairs are classified into confident and unsupported ones according to the GenMiR++ scores. We applied the profile correlation computation to each pair.

### Intronic miRNAs and their host genes

For miRNA genes located within non-coding regions, the expression patterns might be found similar to those mRNAs transcribed from the same protein-coding genes. We therefore collected published information about intronic miRNAs and their host genes [38]. The Pearson correlation coefficients for corresponding probe-probe pairs were then computed as the estimation of correlations.

### Statistical analysis

All data were analyzed with the R software [43]. To test the association between paired miRNA-mRNA profiles, the Pearson correlation coefficients and *p*-values were computed. Because significant results might occur by chance during multiple tests, we adjusted the *p*-values with the Benjamini and Hochberg method to control the false discovery rate [44].

## Authors' contributions

YW collected experimental data and conducted the computational analysis. KL initiated and supervised the project, helped with biological interpretation, drafted and revised the manuscript. Both authors read and approved the final manuscript.

## Supplementary Material

Additional file 1 **Significantly correlated microarray probe pairs**. Significantly correlated microarray probe pairs, predicted by TargetScan 4.1. Pearson correlation coefficients, *p*-values and Benjamini and Hochberg adjusted *p*-values are listed. Click here for file

Additional file 2 **Significantly correlated microarray probe pairs**. Significantly correlated microarray probe pairs, predicted by miRBase. Pearson correlation coefficients, *p*-values and Benjamini and Hochberg adjusted *p*-values are listed. Click here for file

Additional file 3 **Probe-probe correlations for GenMiR++ predicted miRNA-target pairs**. Significantly correlated microarray probe pairs, data taken from GenMiR++. Pearson correlation coefficients, *p*-values and Benjamini and Hochberg adjusted *p*-values are listed. Click here for file

Additional file 4 **Probe-probe correlations for experimentally supported miRNA-target pairs in TarBase**. Significantly correlated microarray probe pairs, data taken from Tarbase version 4. Pearson correlation coefficients, *p*-values and Benjamini and Hochberg adjusted *p*-values are listed. Click here for file

Additional file 5 **Significant correlations between known intronic miRNAs and host genes**. Significantly correlated microarray probe pairs for intronic miRNAs and their host genes. Pearson correlation coefficients, *p*-values and Benjamini and Hochberg adjusted *p*-values are listed. Click here for file

Additional file 6 **Study on spatial biases of the miRNA array design**. To study the spatial biases problem found on microarray design, we performed the autocorrelation analysis on the OSUCCC miRNA microarray. The procedure has been described in [40]. The analysis was applied to the 60 cancer cell lines in MCI-60 miRNA expression profiles. Among which we show four results to illustrate that periodic autocorrelations were not obvious. Click here for file
