# Supplementary material for: Correlation of expression profiles between microRNAs and mRNA targets using NCI-60 data
Source: BMC Genomics. 2009 May 12;10:218. doi: 10.1186/1471-2164-10-218 (PMC2686738; doi:10.1186/1471-2164-10-218)
Supplement: Additional file 6 — Study on spatial biases of the miRNA array design. To study the spatial biases problem found on microarray design, we performed the autocorrelation analysis on the OSUCCC miRNA microarray. The procedure has been described in [40]. The analysis was applied to the 60 cancer cell lines in MCI-60 miRNA expression profiles. Among which we show four results to illustrate that periodic autocorrelations were not obvious. [file 1471-2164-10-218-S6.doc]

### Additional file 6 – Study on spatial biases of the miRNA array design

To study the spatial biases problem found on microarray design, we performed the autocorrelation analysis on the OSUCCC miRNA microarray. The procedure has been described in [40]. The analysis was applied to the 60 cancer cell lines in MCI-60 miRNA expression profiles. Among which we show four results to illustrate that periodic autocorrelations were not obvious.
